# The providing resources to enhance African American patients’ readiness to make decisions about kidney disease (PREPARED) study: protocol of a randomized controlled trial

**DOI:** 10.1186/1471-2369-13-135

**Published:** 2012-10-12

**Authors:** Patti L Ephraim, Neil R Powe, Hamid Rabb, Jessica Ameling, Priscilla Auguste, LaPricia Lewis-Boyer, Raquel C Greer, Deidra C Crews, Tanjala S Purnell, Bernard G Jaar, Nicole DePasquale, L Ebony Boulware

**Affiliations:** 1 Welch Center for Prevention, Epidemiology and Clinical Research, Johns Hopkins Medical Institutions; 2 Department of Epidemiology, Johns Hopkins Bloomberg School of Public Health; 3 San Francisco General Hospital and University of California San Francisco; 4 Division of Nephrology, Johns Hopkins Medical Institutions; 5 Division of General Internal Medicine, Johns Hopkins University School of Medicine; 6 Department of Health Policy and Management, Johns Hopkins Bloomberg School of Public Health; 7 Nephrology Center of Maryland

**Keywords:** Shared decision-making, Live kidney transplantation, Live kidney donation, Chronic kidney disease, End stage renal disease

## Abstract

**Background:**

Living related kidney transplantation (LRT) is underutilized, particularly among African Americans. The effectiveness of informational and financial interventions to enhance informed decision-making among African Americans with end stage renal disease (ESRD) and improve rates of LRT is unknown.

**Methods/design:**

We report the protocol of the Providing Resources to Enhance African American Patients’ Readiness to Make Decisions about Kidney Disease (PREPARED) Study, a two-phase study utilizing qualitative and quantitative research methods to design and test the effectiveness of informational (focused on shared decision-making) and financial interventions to overcome barriers to pursuit of LRT among African American patients and their families. Study Phase I involved the evidence-based development of informational materials as well as a financial intervention to enhance African American patients’ and families’ proficiency in shared decision-making regarding LRT. In Study Phase 2, we are currently conducting a randomized controlled trial in which patients with new-onset ESRD receive 1) usual dialysis care by their nephrologists, 2) the informational intervention (educational video and handbook), or 3) the informational intervention in addition to the option of participating in a live kidney donor financial assistance program. The primary outcome of the randomized controlled trial will include patients’ self-reported rates of consideration of LRT (including family discussions of LRT, patient-physician discussions of LRT, and identification of a LRT donor).

**Discussion:**

Results from the PREPARED study will provide needed evidence on ways to enhance the decision to pursue LRT among African American patients with ESRD.

**Trial registration:**

ClinicalTrials.gov NCT01439516

## Background

African Americans are less likely than Whites to receive kidney transplants, despite their up to four-fold greater likelihood to develop end stage renal disease (ESRD) [1]. Several factors contribute to lower rates of kidney transplantation for African Americans, including immunological incompatibility of deceased donor kidneys [2,3], lower rates of referral of African Americans for transplantation [4-6], lower rates of deceased kidney donation [7], less access to health care [5,8], less desire for kidney transplantation [9,10], and suboptimal discussions about living related kidney transplantation (LRT) between recipients, their families, and health care providers [11]. LRT offers patients an opportunity to bypass many barriers to receipt of deceased kidney transplants, and it improves survival and quality of life at a lesser cost than dialysis care [12-16]. However, African Americans are less likely than Whites to receive LRTs, further exacerbating race disparities in transplant rates [17].

African Americans’ poor awareness of the risks and benefits of LRT as well as their limited access to potential live kidney donors represent important potential barriers to their receipt of LRT [18]. Evidence suggests African Americans are less likely than their White counterparts to be aware of LRT as a treatment option, even when they are under the care of a nephrologist [19,20]. Evidence also suggests African Americans have difficulty identifying potential live kidney donors that successfully complete the donation process [21]. Lack of financial assistance with indirect costs associated with LRT, such as expenses from travel/lodging, lost work, and home health assistance for donors may pose a significant barrier to live kidney donation, particularly among African Americans [22]. A recent study of the general public identified that potential living donors concerned about out of pocket expenses related to donation had 50% lesser odds of being willing to donate when compared to persons not concerned about expenses [23]. In a separate study, an overwhelming majority of the U.S. general public reported having favorable attitudes toward financial assistance programs which could help cover indirect costs for living donors, including costs associated with leave from work, with African Americans reporting significantly more favorable attitudes towards some forms of financial assistance when compared to Whites [24]. To date, however, it is unknown whether educational or financial assistance to address gaps in patients’ knowledge and financial barriers to receipt of LRT could be effective mechanisms of improving African Americans’ access to this life-saving therapy.

We describe the protocol of a two-phase study in which we 1) developed informational and financial interventions to enhance African Americans’ shared and informed decision-making about LRT and 2) are currently testing the effectiveness of these interventions in a randomized controlled trial.

## Methods/design

### Study design summary

The Providing Resources to Enhance African American Patients’ Readiness to Make Decisions about Kidney Disease (PREPARED) Study is a two-phase study to design and test the effectiveness of informational and financial interventions to overcome barriers to pursuit of LRT among African American patients and families. In Phase 1, we developed an informational intervention, including an educational video and handbook, to assist patients with new-onset ESRD and their families with informed decision-making regarding the choice of LRT and other renal replacement therapy options. We also developed a financial assistance intervention for potential live kidney donors identified by patients with ESRD, which we adapted from an existing national program [25]. In Phase 2, we are currently conducting a randomized controlled trial to assess the individual and combined effectiveness of informational and financial interventions on patients’ and their families’ consideration or pursuit of LRT as a treatment. The Johns Hopkins School of Medicine Institutional Review Board has approved all study procedures.

### Phase I: Development of informational and financial assistance interventions

To inform the development of informational interventions, we performed qualitative focus groups as well as directed interviews of patients with kidney disease and their family members. Full details have been previously published [26]. We also performed systematic reviews of scientific evidence describing the risks and benefits of LRT compared to other forms of renal replacement therapy [27-30]. We reviewed the published literature describing ethical and legal forms of potential financial assistance for patients and living donors seeking LRT (e.g. reimbursement for childcare expenses, lodging/travel expenses, and time lost from work) to inform intervention development [31-40]. We also reviewed publicly available information on a national live donor financial assistance program to inform the development of our donor financial assistance intervention [25]. We performed several iterative revisions of informational materials, incorporating feedback from patients with kidney disease and their family members.

### Description of informational intervention

The informational intervention consists of an educational video and written handbook that we designed to assist patients with kidney disease (potential transplant recipients) and their family members (potential donors) with making informed decisions about LRT. The informational materials are intended to expose potential recipients and donors to a variety of experiences of others receiving various forms for renal replacement therapy, including LRT.

The educational video features patients and their family members describing their experiences with LRT as well as with other forms of renal replacement therapy (peritoneal dialysis, home hemodialysis, in-center hemodialysis, and medical management with no dialysis or transplant). The educational video also features medical professionals (a transplant nephrologist, a transplant social worker, a general nephrologist and a dialysis social worker) describing positive and negative experiences patients could have with the various forms of renal replacement therapy. The written handbook provides a summary of scientific evidence describing the risks and benefits of LRT and other forms of renal replacement therapy in lay language. Together, the educational video and handbook promote shared and informed decision-making regarding LRT on the part of patients, their families, and health care providers by achieving commonly accepted goals for informational shared decision-making aids [41].

### Description of the financial assistance intervention

We will offer potential recipients the opportunity to enroll their family members or friends (potential donors) in a program that will reimburse potential donors up to $1600.00 for approved financial assistance through the study by submitting formal original invoices, receipts, and other documentation of their qualifying medical and non-medical expenses related to live kidney donation evaluation, donation, or convalescence (up to 10 weeks post live donor/LRT procedures). Qualifying expenses include travel/lodging, meals, incidental expenses (e.g., parking), lost wages, and childcare costs incurred by the potential donor as part of: (1) donor evaluation, clinic visit or hospitalization, (2) hospitalization for the living donor surgical procedure, and/or (3) medical or surgical follow-up clinic visits or hospitalization within 90 days following the living donation procedure. Participants will be permitted to utilize the $1600.00 for multiple approved purposes, as long as the total reimbursement is not greater than $1600.00. Also, more than one potential donor may draw from these funds, as long as the total value of reimbursed expenses does not exceed $1600.00 per patient study participant. This program covers a broader array of expenses related to live kidney donation than other national programs and does not limit reimbursement of potential donors based on their personal incomes.

### Phase 2: Randomized controlled trial assessing the individual and combined effectiveness of informational and financial interventions

**Figure 1:**
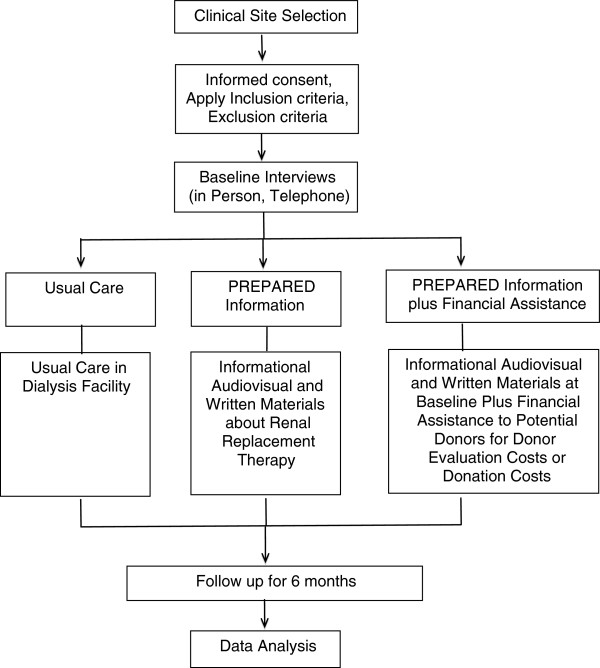
Flow of PREPARED Study Activities.

We are currently conducting a randomized controlled trial to assess the individual and combined effectiveness of informational and financial assistance interventions to enhance rates of LRT. The combined intervention will be tested to detect any additive or multiplicative effects of the financial assistance intervention. Recruitment of study participants began in August, 2012. Figure 1 contains a flow diagram demonstrating the overall structure of our study.

#### Target population and eligibility criteria

Our population includes English-speaking African American patients with ESRD who are age 18 years or older and who have initiated dialysis within one year prior to study enrollment. We exclude participants if they have dementia (assessed in-person) [42] or if they have a self reported history of prior kidney transplant, cancer within 2 years prior to recruitment, stage IV congestive heart failure, end stage liver disease, pulmonary hypertension, severe peripheral vascular disease, or chronic (debilitating) infections. We assess participants’ eligibility via questions administered by a research assistant in-person in dialysis units. After potentially eligible patients agree to participate, we request a release of medical records from each participant to confirm their eligibility for the study and to ascertain whether potential donors have contacted local transplant centers to be evaluated as donors on behalf of participants.

#### Recruitment of participants

We recruit participants from academically affiliated and community-based dialysis centers in the Baltimore, MD metropolitan region, which we have selected to provide an adequate number of African American participants with new-onset ESRD. We recruit patients on-site from different dialysis centers on different days. We systematically rotate recruitment days at sites so variations in patient scheduling at all sites can be covered to achieve the maximum number of recruitment opportunities. Upon confirmation of their eligibility to participate, we randomly assign patients into intervention groups.

#### Reimbursement and enrollment of potential kidney donors

Potential kidney donors only become aware of the financial assistance program through direct referral by enrolled study participants. Research study staff do not contact or attempt to contact any family, friends, or acquaintances of enrolled patient participants. Therefore, enrolled participants will be directed to contact study staff in the event they want to refer potential donors to the program. To be eligible to participate, potential donors must speak English and be 18 years or older. Once potential donors contact study staff, study staff arrange for a time to meet with the potential donor(s) in person and mail them the brochure describing the financial assistance program. At that time, study staff explain the financial assistance program to potential donors, including what they will be asked to do as part of the study. Potential donors are notified that participation in the financial assistance program offered by the study may preclude them from receiving financial assistance from any other program (e.g., National Living Donor Assistance Center). If potential donors wish to participate in the financial assistance program, we obtain consent for their participation in the study using approved consent procedures.

#### Randomization

Using blind and secure allocation by computer, we randomly assign participants to one of three intervention arms: 1) control group (“Usual Care”, N=70); 2) the informational intervention (“PREPARED”, N=70); and 3) the informational plus the financial assistance interventions (“PREPARED plus Financial Assistance”, N=70). Randomization is blocked by recruitment site to ensure equal allocation within each dialysis facility.

#### Usual care group

Participants randomly assigned to receive Usual Care proceed with their clinical care in dialysis centers as already routinely implemented by their physicians. At study conclusion, participants will receive informational materials provided in PREPARED (educational video and written handbook) after follow up assessments have been completed.

#### Intervention groups

Participants randomly assigned to the PREPARED program receive the informational intervention, which consists of an educational video and written handbook intended to be used together to inform patients’ decisions about their kidney disease treatment choices. These materials are provided to participants on a day when they do not receive dialysis in the dialysis center. Participants meet with trained research study staff during which time they are oriented to the book and watch the video. Study staff members also encourage participants to share the materials with their families and health care providers. Participants randomly assigned to the PREPARED plus Financial Assistance program receive the informational intervention at the time of enrollment in a similar fashion with a similar approach to the PREPARED assignment. In addition, participants in this group are invited to participate in the live kidney donor financial assistance program as described above.

### Data collection, follow-Up and outcomes

#### Measures assessed through interventions

All patient participants enrolled in the study are assessed using an in-person questionnaire at the time of recruitment and a structured telephone interview at baseline, 1 month, 3 months, and 6 months. Trained study staff administer all telephone interviews. For the informational and financial intervention arms, participants are contacted via telephone to determine whether they have viewed the materials provided to them (an educational video and written handbook for the informational intervention and the brochure for the financial intervention arm) again at home. Participants are also asked if they have shared or discussed the content of intervention materials with family members.

The primary outcome of interest is patients’ pursuit of LRT over six months of follow up. This outcome is measured as participants’ achievement of at least one of six key steps toward pursuing LRT, including: 1) execution of patient-family discussions about LRT; 2) execution of patient-physician discussions about LRT; 3) initiation of the recipient evaluation for LRT; 4) completion of the recipient evaluation for LRT, 5) identification of a potential live kidney donor, and 6) being listed at a transplant center. Pursuit of LRT will be assessed via questionnaires administered via telephone questionnaire at baseline, 1 month, 3 months and 6 months follow-up time periods. For participants reporting being registered on a transplant waiting list, we will contact transplant centers to determine whether there have been any live donor inquiries on behalf of the participant. We will also assess whether patient participants receive LRT and whether patient participants’ family members inquire about or use the financial assistance program (for those randomized to the PREPARED plus Financial Assistance arm).

Other measures include assessment of participants’ (1) knowledge of LRT; (2) interest in LRT; (3) perceived barriers to LRT; (4) preferred role in decision-making about LRT; (5) concerns about the risks of LRT to themselves and to donors [43-45]; (6) family structure and relationships [46]; (7) interest in other forms of renal replacement therapy (i.e., peritoneal dialysis or home-hemodialysis); (8) satisfaction with hemodialysis therapy; (9) depression and social support [47,48]; (10) financial stress [49]; (11) health literacy [50] and numeracy [51,52]; (12) trust in the medical system [53,54], (13) comorbid conditions [55] and (14) sociodemographic characteristics. For patient participants assigned to the PREPARED and PREPARED plus Financial Assistance groups, we also assess their perceptions of the helpfulness of the video and handbook. In all study groups, we also assess the number of donor inquiries on behalf of study participants at local transplant centers (Table 1).

**Table 1:** Data collected at study baseline and follow up

	**PATIENT**			
**Measure**	**Baseline**	**1**	**3**	**6**
		**Month**	**Month**	**Month**
**Current Treatment Information**				
Date and Place of Initiation of Dialysis	X			
Presence of AV^*^ Fistula or AV Graft	X			
Presence of Peritoneal Catheter	X	X	X	X
**Exposure to Information about Kidney Disease Treatment**				
In Center Hemodialysis Information	X	X	X	X
Home Hemodialysis Information	X	X	X	X
Peritoneal Dialysis Information	X	X	X	X
Kidney Transplant Information	X	X	X	X
Live Donor Kidney Transplant Information	X	X	X	X
**Discussions with Health Care Providers about Kidney Disease Treatment**				
Occurrence of Physician Discussions-Nephrologists	X			
Satisfaction with Patient-Physician Discussions	X			
Physician Recommendations Regarding LRT*	X			
**Belief & Knowledge about Treatment for Kidney Failure**, **Interest in LRT**				
Beliefs About Treatment for Kidney Failure	X	X	X	X
Knowledge of LRT	X	X	X	X
Interest in LRT	X	X	X	X
Consideration of LRT (stage placement)	X	X	X	X
**Elements of Shared Decision Making**				
Stage in Decision Making [43]	X	X	X	X
Decision Self-Efficacy [43]	X	X	X	X
Decisional Conflict Scale [44]	X	X	X	X
Interest in Switching Renal Replacement Therapy				
**Pursuit of LRT**, **Barriers to Completing Behavior Stages**				
Quality of Family Discussion	X	X	X	X
Information on Donor	X	X	X	X
Barriers to Patient-Family Discussion	X	X	X	X
Barriers to Patient-Physician Discussion	X	X	X	X
Barriers to Starting Evaluation	X	X	X	X
Barriers to Completing Evaluation	X	X	X	X
**Mediators and Correlates of Pursuit of LRT**				
Trust in Medical Care [53,54]	X			
In-Center Dialysis Care - Nephrologist Communication, Receipt of Information	X	X	X	X
Family Functioning [46]	X			
Depressed Mood PRIME-MD/PHQ^*^[48]	X		X	X
Social Function - Emotional Support, Instrumental Support, Informational Support, Social Isolation [47]	X			
Personal Financial Well-being Scale [49]	X			
Charlson Comorbidity Index [55]	X			
Sociodemographic Information	X			
**Assessment of Book and Video**				
Assessment of Book and Video		X	X	X
**Health Literacy and Numeracy**				
Rapid Estimate of Adult Literacy in Medicine (REALM) [50]	X			
Subjective Numeracy and Risk Numeracy [51,52]	X			

*Abbreviations are as follows: living related kidney transplantation (LRT); arteriovenous (AV); primary care evaluation of mental disorders/patient health questionnaire (PRIME-MD/PHQ).

### Statistical considerations

#### Data analysis

Randomly assigned intervention group (i.e. Usual Care, PREPARED, PREPARED plus Financial Assistance) will be the main independent variable for intent-to-treat analyses. In our primary analyses, we will assess the proportion of participants in each group achieving at least one new LRT pursuit behavior over 6 months. We will use multivariable models (e.g. logistic regression) to assess differences in the odds of achieving LRT pursuit behaviors among study groups while adjusting for study site characteristics and other baseline characteristics found not to be balanced by randomization. Secondary analyses will include exploratory analyses among persons within subgroups defined post-randomization--for example, analyses among persons with greater versus lesser knowledge of LRT at baseline. We will conduct primary analyses under the assumption that data is missing at random, however, we will also perform sensitivity analyses based on other scenarios (i.e. patterns of missing data) to evaluate the robustness of our assumptions.

#### Sample size and power

We are aware of no other randomized controlled trials evaluating the effectiveness of informational (focused on shared decision-making) or financial interventions on commitment to LRT in incident dialysis patients. In our own work, an educational intervention resulted in patients’ greater pursuit of LRT (30% achieving new LRT behavior in Usual Care arm compared to 50% in education arm over 6 months) [19]. Our own previous work also demonstrated African Americans in the US general public were supportive of live kidney donors receiving reimbursement for expenses related to donation [24]. We anticipate the financial intervention, which addresses a common and tangible barrier to live kidney donation, may improve pursuit of LRT by an additional 10%. Based on these assumptions, we will have over 80% power to detect a trend of increasing pursuit of LRT across groups (e.g., 30% in Usual Care, 50% in PREPARED group, 60% in PREPARED plus Financial Assistance group) at 6 months. We will also have over 80% power to detect a 30% difference between the PREPARED group and the Usual Care group or the PREPARED plus Financial Assistance group and the Usual Care group individually with correction for multiple comparisons.

## Discussion

Inadequate resources to promote shared decision-making about LRT and financial barriers to live kidney donation may contribute to lower rates of LRT among African American compared to Whites [56-58]. We designed PREPARED educational interventions to directly address commonly encountered barriers to African American patients and their family members and health care providers in engaging in informed and shared decision-making regarding pursuit of LRT. We designed the PREPARED plus Financial Assistance intervention to enable patients and their families to overcome the significant barrier of monetary concerns in pursuit of LRT. Rigorous study of these interventions will provide health care providers and policy makers with the evidence necessary to endorse the widespread use of similar interventions in a variety of clinical settings if they are effective.

## Abbreviations

ESRD: End stage renal disease; LRT: Living related kidney transplants or transplantation, PREPARED, Providing Resources to Enhance African American Patients’ Readiness to Make Decisions about Kidney Disease.

## Competing interests

The authors do not declare any competing interests.

## Authors’ contributions

PLE contributed to the study design, intervention development, study conduct, and drafting and approval of the manuscript. NRP contributed to the study conception and design, intervention development, study conduct, and drafting and approval of the manuscript. HR contributed to the study conception and design, intervention development, study conduct, and drafting and approval of the manuscript. JA contributed to the intervention development and drafting and approval manuscript. PA contributed to the intervention development and drafting and approval of the manuscript. LLB contributed to the intervention development and drafting and approval of the manuscript. RCG contributed to the intervention development and drafting and approval of the manuscript. DCC contributed to the intervention development and drafting and approval of the manuscript. TSP contributed to the intervention development and drafting and approval of the manuscript. BGG contributed to the intervention development and drafting and approval of the manuscript. ND contributed to the intervention development and drafting and approval of the manuscript. LEB led the study conception and design, intervention development, study conduct, and drafting and approval of the manuscript. All authors read and approved the final manuscript.

## Pre-publication history

The pre-publication history for this paper can be accessed here:

http://www.biomedcentral.com/1471-2369/13/135/prepub
